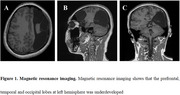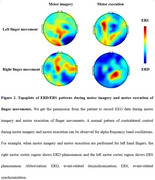# One hemisphere can succeed in normal cognition and daily functioning: a case report

**DOI:** 10.1002/alz70857_100890

**Published:** 2025-12-24

**Authors:** Xiaoduo Liu, Zhibin Wang, Yi Tang

**Affiliations:** ^1^ Xuanwu Hospital, Capital Medical University, Beijing, Beijing, China; ^2^ Department of Neurology & Innovation Center for Neurological Disorders, Xuanwu Hospital, Capital Medical University, National Center for Neurological Disorders, Beijing, Beijing, China; ^3^ Neurodegenerative Laboratory of Ministry of Education of the People's Republic of China, Beijing, Beijing, China

## Abstract

**Background:**

Traumatic brain injury is a leading source of childhood injury that leads to disability. The extent of neuroplasticity that help to adapt and recover after brain injury is not fully understood. The present case describes an adult with severe intracranial hemorrhage due to head trauma at 2 months after birth with underdevelopment of the prefrontal, temporal and occipital lobes at left hemisphere. Surprisingly, this patient presents a normal cognitive and motor functioning which guarantees a stable job.

**Method:**

A 29‐year‐old female was referred to our memory clinic for consultation related to a chief complain of transient memory loss at 3 months ago. At the morning after she got up, she suddenly could not remember her workplace and work schedule. Approximate 3–5 minutes later, she gradually regained her memory and went to work. Other neurological symptoms had not been observed during the episode. She reported a history of traumatic brain injury at 2 months after birth.

**Result:**

Clinical examinations were performed for differential diagnosis. Magnetic resonance imaging shows that the prefrontal, temporal and occipital lobes at left hemisphere was underdeveloped (Figure 1), while other abnormal signals were not detected in T1, T2, FLAIR, and DWI images. Computed tomography angiography of cerebral vessels does not find prominent narrowing or malformation. Electroencephalogram of 20 minutes recording does not detect any epileptic waveforms. Laboratory tests (e.g., antinuclear antibody, antiphospholipid antibody, coagulation, etc.) are not significant. Neuropsychological assessment shows normal scores. Considering the absence of sufficient evidences that support stroke, seizures, migraine and other potential causes, a diagnosis of transient global amnesia therefore had been made. Education regarding to the benign nature of this condition were provided to the patient and her families, and attention on recurrent episodes were recommended. No recurrence was observed at the two‐month visit.

**Conclusion:**

This individual with underdevelopment of left hemisphere due to traumatic brain injury at infancy presents normal cognition and motor functioning (Figure 2) at adulthood, which enable a normal life with a stable job. This case indicates that human brain has a strong capacity of neuroplasticity adapting to adverse clinical condition, which may guide rehabilitation and therapies.